# A Dual Case of Peritonitis and Central Nervous System Infection Caused by Nutritionally Variant Streptococcal Species

**DOI:** 10.1155/2017/6012964

**Published:** 2017-01-23

**Authors:** Sussi Vivar, Jennifer E. Girotto, Thomas S. Murray

**Affiliations:** ^1^Connecticut Children's Medical Center, Hartford, CT, USA; ^2^School of Pharmacy, University of Connecticut, Storrs, CT, USA; ^3^Frank H. Netter MD School of Medicine, Quinnipiac University, Hamden, CT, USA

## Abstract

Nutritional variant streptococci (NVS) are difficult to identify bacteria that can cause invasive infections such as endocarditis and meningitis. NVS as a cause of peritonitis has not been routinely described. This case of NVS as the etiology of peritonitis associated with previous neurosurgery and ventriculoperitoneal (VP) shunt revision demonstrates its potential role as a significant pathogen in patients with peritonitis and VP shunts. Therapy consists of vancomycin plus a second agent but since there are no standards for susceptibility testing, clinical response remains the standard for determining the efficacy of treatment. When there is central nervous system (CNS) involvement it is important to include drugs with appropriate CNS penetration.

## 1. Introduction

Nutritionally variant streptococci (NVS) comprise the* Abiotrophia* sp. and* Granulicatella* sp. and are normal residents of the oral cavity, gastrointestinal, and urogenital tracts [1, 2]. Given that NVS are fastidious, grow slowly, and can be pleiomorphic on Gram stain, these bacteria may be misidentified [1]. NVS causes a variety of invasive infections, most commonly endocarditis as a result of bacteremia [2]. Other reported infections include osteomyelitis, otitis media, wound infections, septic arthritis, and pancreatic abscesses and infections of the CNS [2–4]. Here we describe a case of CNS shunt associated peritonitis where the cultures from the CNS grew pure NVS. While peritonitis is a well-known complication of VP shunt infections, this organism as a pathogen in significant abdominal infections in children is rarely reported.

## 2. Case Presentation

A 13-year-old girl with hydrocephalus managed with bilateral ventriculoperitoneal (VP) shunts presented to the Emergency Department (ED) with 24 hours of worsening generalized abdominal pain, headaches, dizziness, and lethargy without fever. She was born prematurely at 27 weeks of gestation resulting in developmental delay and her adoptive parents provided the history. Her parents reported she had decreased oral intake for four days prior to presentation with chills but no fever, emesis, or urinary symptoms. She had 20 VP shunt revisions in her lifetime, the most recent being completed two months prior to presentation. Her additional past medical history was significant for epilepsy, chronic headaches, cerebral palsy, systemic lupus erythematosus, constipation, and gastroesophageal reflux disease. Her medication list included Fioricet, eletriptan, prednisone, hydrochloroquine, lansoprazole, ranitidine, phenobarbital, and polyethylene glycol.

In the ED she was ill-appearing, uncomfortable, and actively complaining of abdominal pain. Her vital signs were as follows: blood pressure 123/93, heart rate 126 beats/min, temperature 37.6°C, respiratory rate of 24/min, and pulse oximetry of 100% on room air. Her physical exam showed that her lung sounds were clear to auscultation and the heart rate was sinus tachycardia with regular rhythm without murmur on auscultation. Her abdomen was distended and diffusely tender without guarding or rebound.

**Figure 1:**
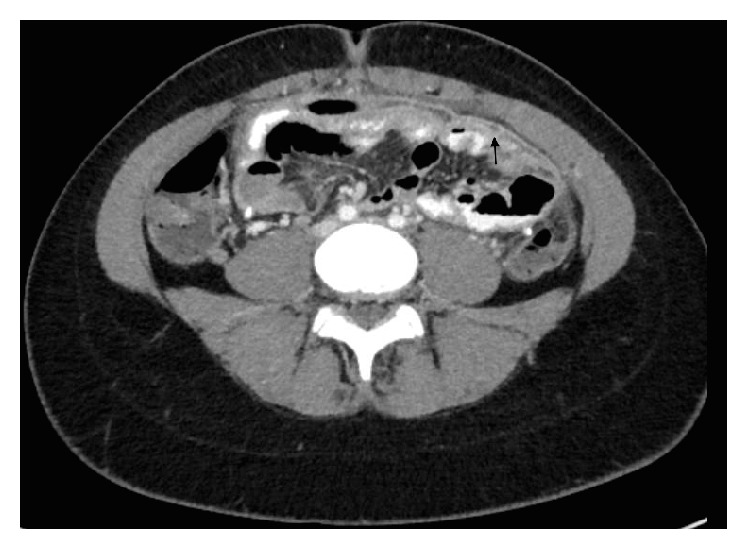
Peritonitis due to NVS. Abdominal CT scan with contrast. The arrow points to the hyperintensity demonstrating fluid accumulation consistent with peritonitis.

Initial laboratory values were as follows: white blood cell count 10,000 cells/uL (normal 4.5–13 × 10^3^/uL) with 78.5% neutrophils (normal 34–64%), 11.3% lymphocytes (normal 35–45%), 0.2% eosinophils (normal 0–3%), hemoglobin concentration 10.8 g/dL (normal 12–16 g/dL), platelets 289,000/uL (normal 150–450 × 10^3^/uL), C-reactive protein (CRP) 25.2 mg/dL (normal < 1.0 mg/dL), and gamma-glutamyl transferase 349 U/L (normal range 7–32 U/L). Alanine aminotransferase and aspartate aminotransferase values along with amylase and lipase levels were within normal limits. A CT scan of the head with and without contrast showed stable appearance of shunted hydrocephalus. An abdominal CT scan performed with oral and intravenous contrast showed moderate pelvic-free fluid with peritoneal enhancement and mild to moderate diffuse thickening of the adjacent ileum and terminal ileum (Figure 1). The two ventriculoperitoneal shunt tubes were observed with no evidence of loculated fluid around the tip of either shunt.

The presence of abdominal pain, significantly elevated CRP, and accumulation of peritoneal fluid on CT scan were consistent with acute peritonitis and she was admitted to the Pediatric Intensive Care Unit for further care. She underwent laparoscopic surgery and VP shunt externalization within a few hours of admission where peritoneal cavity and cerebral spinal fluid (CSF) were collected for bacterial culture. A blood culture was not obtained at admission as she was afebrile with a normal white blood cell count and NVS was not initially suspected as pathogen. Empiric antibiotic therapy was started with vancomycin, piperacillin/tazobactam, and metronidazole. The Gram stain of both the CSF and peritoneal fluid showed Gram positive cocci. Despite antibiotic therapy, she developed fevers as high as 39.1°C on the third day of admission.

**Figure 2:**
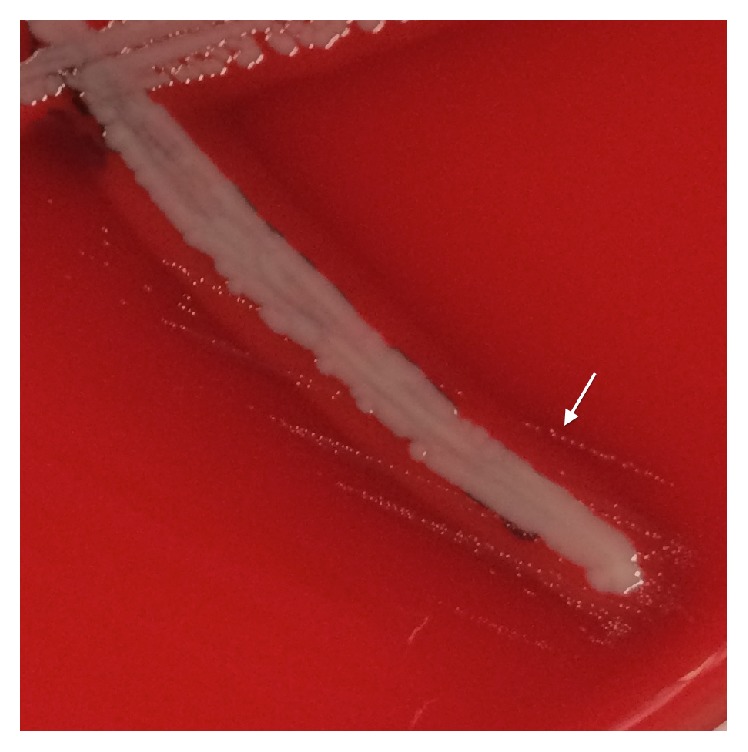
Satellite growth of nutritional variant streptococci (NVS) around* Staphylococcus aureus*. Small colonies of NVS are seen in areas on the blood agar plate where* S. aureus* has completely lysed red blood cells (*β*-hemolysis), releasing nutrients required for NVS growth.

Both CSF and peritoneal cultures were positive on day 3 with faint bacterial growth on the chocolate agar plates. Growth on blood agar plates occurred only in the presence of *β*-hemolytic* Staphylococcus aureus* (Figure 2) and the organism was identified as NVS species. All specimens from the peritoneum and CSF grew pure NVS without other bacteria. The laboratory was unable to determine the antibiotic susceptibilities as it could not recover viable organism under conditions that would permit routine susceptibility testing or further identification of the organism at the genus level.

Based on the culture results, rifampin was added 300 mg every 12 hours (adult dose) and the vancomycin was continued (600 mg (15 mg/kg/dose) every 8 hours to maintain a trough of 15–20 mg/L). Piperacillin/tazobactam was discontinued but the primary team remained concerned about anaerobic bacteria not recovered via standard culture techniques so they continued metronidazole (400 mg every 6 hours (40 mg/kg/day)). CSF cultures collected on a daily basis for the next 7 days after the initial positive cultures remained with no growth demonstrating a rapid clearance of her CNS infection. She defervesced, her abdominal pain improved, and the recommendation was made to convert her shunt system to a ventriculoatrial (VA) shunt 2-3 weeks after sterilization of the central nervous system. A blood culture taken on approximately day 12 of hospitalization was negative prior to shunt revision and she had three additional negative blood cultures during antibiotic therapy. She was treated with rifampin for a total of 11 days and originally scheduled for four weeks of vancomycin therapy but this was extended to 12 weeks until her CRP normalized which would be a sufficient length of therapy for endocarditis. However, she continued to have no murmur on cardiac auscultation and an echocardiogram was not performed. One year later she still has the VA-shunt in place and has not had any subsequent positive blood cultures, documented peritonitis, CNS infection, or signs and symptoms of clinical relapse.

## 3. Discussion

There is great variety in the types of Gram positive and Gram negative organisms that can cause peritonitis. In the presented case, the Gram positive organisms seen on staining of the CSF and peritoneal fluid grew poorly in the clinical microbiology laboratory, suggesting this was not a common pathogen. Satellite growth in the presence of* S. aureus* confirmed a nutritionally fastidious organism (Figure 2). In fact, NVS should be considered when the Gram stain of the clinical specimen reveals Gram positive cocci in chains consistent with streptococci that grow poorly or not at all on routine culture. These bacteria do not grow well on agar media apart from hematin and grow best with the addition of PHC pyridoxal hydrochloride which provides the bacteria with vitamin B6 necessary for their growth [2].

There is one report of peritonitis in an adult patient with end stage renal disease where the peritoneal cultures, like this case, grew pure NVS (*Abiotrophia* sp.) [5]. The majority of NVS of the CNS are associated with either endocarditis, or a recent neurosurgical procedure including one case of a patient with recent VP shunt revision [5–8]. By the time this organism was identified in this patient she was on antibiotic therapy and improving so an echocardiogram was not performed and her infection was attributed to her recent VP shunt revision. However, these previously reported CNS infections have not been associated with peritonitis. Interestingly, this patient's last shunt revision was done less than two months prior to presentation.

Very little is known about the microbial pathogenesis of NVS. Okada et al. studied 17* Abiotrophia* strains from the oral cavity of healthy patients and seven from patients with proven endocarditis in a rat model of endocarditis [9]. All seven* Abiotrophia* strains that caused endocarditis in people were highly infective in the rat model. Of the strains from healthy controls, the infectivity appeared to be species dependent with* A. adiacens* showing the highest rates of infectivity and* A. para-adiacens* and* A. elegans* the lowest [9]. Interestingly, the highly infective* A. adiacens* strains tended to show markedly higher fibronectin-binding capacity compared with less infective strains, suggesting a possible relationship between the fibronectin-binding capacity and ability to cause disease [9]. In this case, one possibility is that the NVS attached to and colonized the VP tubing in the subsequent weeks after the surgical revision and this contributed to CSF infection that seeded the peritoneal cavity resulting in peritonitis.

The Infective Endocarditis treatment guidelines recommend aggressive treatment with either a penicillin (i.e., intravenous ampicillin or aqueous crystalline penicillin G sodium) and intravenous gentamicin or the use of intravenous vancomycin and gentamicin [10]. There are no Clinical Laboratory Standard Institute guidelines for antibiotic susceptibility testing for NVS so the ideal antimicrobial regimen is unclear with at least one report of* Abiotrophia* with a vancomycin minimum inhibitory concentration (MIC) of 2-3 mg/L. [5] Further, the endocarditis guidelines state that even if susceptibility is determined, it may be inaccurate and not correlate with clinical cure [11]. Literature reports of small case series show that most strains are thought to be susceptible to vancomycin, gentamicin, and rifampin and that combination therapy may be advisable to prevent relapse [2, 5]. In case series and reports of NVS infections of the CNS, the patients were treated with a beta-lactam or vancomycin as primary therapy with gentamicin and/or rifampin added on for synergy [5–7]. The choice of these agents is consistent with recommendations in the Infective Endocarditis guidelines where systemic concentrations are easy to achieve, but this does not guarantee disease eradication [10, 11].

In cases of CNS infection such as this, appropriate penetration of antibiotics into the CSF at therapeutic levels is essential. Therefore, vancomycin was dosed aggressively to achieve trough concentrations of 15–20 mg/L. Further, rifampin rather than gentamicin was employed because intravenous gentamicin has not demonstrated detectible CNS concentrations beyond the neonatal period [12]. Instead, rifampin with approximately 10% CNS penetration was used to provide synergistic activity. However, no appropriately powered clinical trial has compared various treatment options to determine optimal therapy so clinical response and careful follow-up are currently the best way to determine if the chosen antibiotic regimen is working.
